# Factors and Functions Associated With Health and Well-Being Among Older Adults: Evidence From NHATS

**DOI:** 10.1093/geroni/igab046.743

**Published:** 2021-12-17

**Authors:** Loretta Anderson, Alexandra Wennberg, Allison Gibson

## Abstract

The National Health and Aging Trends Study (NHATS) is a nationally representative sample of Medicare beneficiaries aged 65 and older. From 2011 through 2020, annual in-person interviews have collected data in many areas, including health, environment, wellbeing, cognition, and function. With a decade of follow-up, including replenishment samples, NHATS is an ideal setting to investigate trends and trajectories of aging. Aging is heterogeneous and understanding the myriad of factors and functions that impact health and wellbeing is critical to developing interventions and care to promote health and wellbeing. Considering a multifactorial, wholistic approach to aging will provide a deeper understanding to create an impact. This symposium features pivotal research conducted using NHATS data, while highlighting overall strengths of the dataset for future research. The first presentation of this symposium investigates the factors that define cognitive profiles associated with dementia diagnosis over a period of five years. The second presentation investigates the role engagement in personally meaningful activities play in cognitive, emotional, functional, and health-related outcomes in older adults. The third presentation investigates the association between sleep medication use and fall risk among older adults with and without dementia. The session concludes with an investigation of end-of-life communication in persons with dementia and hearing impairment.

